# Recommendations for Medical Students Completing Virtual Rotations: Lessons Learned from Our Experience During the COVID-19 Pandemic

**DOI:** 10.15694/mep.2021.000036.1

**Published:** 2021-02-04

**Authors:** Caleb Haley, Ethan Song, Samuel Lance

**Affiliations:** 1 University of Michigan Medical School; 2 Morsani College of Medicine; 3 Division of Plastic Surgery

**Keywords:** Education, COVID-19, Medical student education, Virtual, Away rotations

## Abstract

This article was migrated. The article was marked as recommended.

The COVID-19 pandemic prevents medical students who are applying for residency during the 2020-2021 application cycle from completing away rotations. One solution to address this education gap is the creation of virtual sub-internships. The authors provide recommendations to other medical students completing these novel rotations, as well as encourage residency programs to create virtual education opportunities for interested students.

## Introduction

Medical education has faced drastic challenges due to the social distancing efforts from the COVID-19 pandemic. In March 2020 the Association of American Medical Colleges recommended the cancelation of in-person clinical rotations for medical students (AAMC, 2020). Additionally, the Coalition for Physician Accountability recommended that away rotations be discouraged for medical students applying during the 2020-2021 residency application cycle (Coalition for Physician Accountability, 2020). These changes may be especially worrisome for applicants interested in competitive specialties where away rotations are traditionally utilized by nearly all applicants with the goal of increasing their chance of matching at and learning about specific programs (Higgins *et al.*, 2016).

Although challenging, these cancelations provide the opportunity for creative solutions that may improve the equity of the match process (Patel *et al.*, 2020). One such solution has been the creation of virtual sub-internships where students participate in activities such as virtual rounds, case reviews, suturing skills sessions, and educational conferences (Dean *et al.*, 2020). However, these are novel experiences that can be difficult to navigate for both programs and applicants alike.

As two students who recently completed one of the first virtual away rotations at the University of California San Diego Division of Plastic Surgery, we provide the following recommendations to help other students as they embark upon these new educational experiences.

## Recommendations

1. ***Expectation management*** *:* Students should review expectations with the faculty and residents early, so they do not have to clarify them throughout the experience. Some topics include scheduling responsibilities or learning objectives. Students treat these experiences the same as they would an in-person rotation - any expectations that pertain to in-person rotations should also apply to virtual rotations. Students should do their best and show what they can contribute. They should ask for feedback from both the faculty and residents when appropriate. It is important to be engaged and participate; it is okay to be wrong, but make sure to learn from mistakes.
2. ***Scheduling*** *:* Scheduling is perhaps the greatest challenge of a virtual rotation. Students should make sure to review all scheduling documents, calendars, and emails to confirm when activities are scheduled. If there is uncertainty when a meeting is taking place, students should reach out to the residents the day before to confirm the time and obtain any video conferencing information. Also, students should double check the time of events, taking into consideration the time zone conversion if applicable. Providing a phone contact is often a helpful resource in the event of last-minute scheduling changes.
3. ***Teaching sessions*** *:* Didactic discussions and case reviews are often based on the understanding that students have a fundamental grasp of the material and thus take a deeper dive into the nuances. Students should be actively involved in pre-reading and prepare for meaningful discussion during these sessions. Faculty and residents may provide information regarding preferred study resources early in the rotation to enhance this preparation. Students should try to access these resources and use them throughout their rotation.
4. ***Research*** *:* Virtual rotations may include exposing students to the research done at the rotation's institution. Students should ask if they are interested in getting involved with any of the projects. This can be challenging, as most virtual students will not have access to the institution's electronic health record system. However, students can often participate in various non-clinical elements of research for many projects.
5. ***Technical skills sessions*** *:* Surgical specialties may include the opportunity to participate in virtual technical skills sessions, such as suturing workshops. This is best done by setting up another device focused on the technical skills station in addition to the device focused on the student. This will enhance the simulated face-to-face interactions and teaching opportunities of these sessions. Students should practice these technical skills outside of the proctored sessions to ensure technical skill development during the rotation.
6. ***Presentation skills*** *:* Presentations during a virtual sub-internship is a critical opportunity for students to exhibit their communication skills and knowledge. Choosing topics that are of interest to the student will enhance the quality of the presentations. Good topics include personal research, interesting cases, and clinical questions. Students should practice their presentations beforehand, including the timing, transitions, slide animations, and presentation mode. Students should start by introducing themselves, what medical school they attend, and the title of their presentation. Given the virtual format of these presentations, it is very important to speak with appropriate pace, volume, and enunciation using interesting visuals throughout the presentation. Students should look at the camera, not at the presentation on their screen or their notes. Students should end by thanking the program for the opportunity to speak and ask if there are any questions. If the student doesn't know the answer to a question, that's okay. They should admit they are unsure of that question specifically but try to provide a meaningful answer about what they do know. It is a good idea for the student to provide follow-up answers to questions once they have found the right answer.
7. ***Digital professionalism*** *:* As stated earlier, students should behave professionally as if it were an in-person rotation. Students should avoid being too casual, but at the same time try to let the program get to know them. Students should dress just like they would in-person, business professional (although a white coat is most likely not necessary), unless otherwise specified. Students should avoid busy-patterned clothes as these can create a Moiré effect that is visually disturbing. Students should set up a workspace where there are limited distractions and background noise. This includes ensuring students' desks and backgrounds are clean, and silencing all device notifications. It is also important to check all software and equipment ahead of time and optimize their video, lighting, and audio. The camera should be directed at eye level, not pointed up or down towards the student. The student's face should be centered in the video screen, avoiding bright light sources directly behind or in front of them - this can create backlighting and glare. Aim to have gentle light sources coming from multiple locations in the room if possible. Depending on their devices, it may be a good idea to use headphones with an attached microphone for optimal audio. At the beginning of the call, students should introduce themselves if there is someone new on the call. Students should not eat during the call, avoid fidgety behaviors, and minimize unnecessary gestures. If students have technical difficulties, they should assume the people on the other side of the call can still hear and see them and make sure to remain calm. Before the student signs-off, he or she should make sure to thank the residents and faculty for their time.

Lastly, many recent publications have provided medical students and residents with advice for completing virtual interviews, as traditional, in-person interviews have been cancelled due to the COVID-19 pandemic (McKinley *et al.*, 2020; Jones and Abdelfattah, 2020). Much of this advice also applies to virtual sub-internships and we encourage students to review these resources in preparation for their virtual rotations, in addition to their upcoming virtual interviews. 

## Conclusions

The cancelation of away rotations due to the COVID-19 pandemic creates challenges for medical students applying during the 2020-2021 residency application cycle, especially those applying to competitive specialties. However, it also allows for creative solutions including virtual rotations that may improve the equity of the match process as it obviates the cost of travel and housing. We hope that the above recommendations garnered through lessons learned during our initial experience with a virtual away rotation can help other medical students as they complete future virtual rotations as well as future virtual interviews. Similarly, we encourage more residency programs to consider creating virtual rotation opportunities, as they are not only excellent learning experiences for students, but also can assist programs showcase their unique features and get to know prospective applicants. Finally, although virtual rotations are new educational experiences created out of necessity due to the COVID-19 pandemic, we believe these rotations may continue to be beneficial for students and programs alike even once the pandemic has subsided. Virtual rotations are an innovative, logistically flexible, and cost-effective method of engaging students that may reduce disparities by making away rotations more accessible for many students.

## Take Home Messages

- Medical students are not able to complete away rotations due to the COVID-19 pandemic
- One solution to this education gap is the creation of virtual sub-internships
- We provide recommendations to students completing these novel rotations based on our experience completing a virtual sub-internship
- We also encourage residency programs to create virtual opportunities for students
- Virtual rotations may continue after the COVID-19 pandemic subsides because they increase the accessibility of away rotations for more students

## Notes On Contributors

**Mr. Caleb Haley** is a medical student at the University of Michigan Medical School in Ann Arbor, Michigan, USA. ORCID ID: https://orcid.org/0000-0002-8453-0134

**Mr. Ethan Song** is a medical student at the University of South Florida Morsani College of Medicine in Tampa, Florida, USA. ORCID ID: https://orcid.org/0000-0001-8907-904X

**Dr. Samuel Lance** is an assistant professor of surgery at the University of California San Diego Division of Plastic Surgery in San Diego, California, USA. ORCID ID: https://orcid.org/0000-0002-5186-2677

## Bibliography/References

AAMC. (2020) *Important Guidance for Medical Students on Clinical Rotations During the Coronavirus (COVID-19) Outbreak. *Available at: https://www.aamc.org/news-insights/press-releases/important-guidance-medical-students-clinical-rotations-during-coronavirus-covid-19-outbreak (Accessed: 15 July 2020).

Coalition for Physician Accountability. (2020) *Final Report and Recommendations for Medical Education Institutions of LCME-Accredited, U.S. Osteopathic, and Non-U.S. Medical School Applicants*. Available at: https://mk0nrmp3oyqui6wqfm.kinstacdn.com/wp-content/uploads/2020/05/2020.05.06-Final-Recommendations_Final.pdf (Accessed: 15 July 2020).

Dean, R. A., Reghunathan, M., Hauch, A., Reid, C. M., *et al. (*2020) 'Establishing a Virtual Curriculum for Surgical Sub-Internships', *Plast Reconstr Surg*. https://doi.org/10.1097/PRS.0000000000007267

Higgins, E., Newman, L., Halligan, K., Miller, M., *et al. *(2016) 'Do audition electives impact match success?', *Medical Education Online,* 21, pp. 31325-31325. https://doi.org/10.3402/meo.v21.31325

Jones, R. E. and Abdelfattah, K. R. (2020) 'Virtual Interviews in the Era of COVID-19: A Primer for Applicants', *J Surg Educ,* 77(4), pp. 733-734. https://doi.org/10.1016/j.jsurg.2020.03.020

McKinley, S. K., Fong, Z. V., Udelsman, B. and Rickert, C. G. (2020) 'Successful Virtual Interviews: Perspectives From Recent Surgical Fellowship Applicants and Advice for Both Applicants and Programs', *Ann Surg*. https://doi.org/10.1097/SLA.0000000000004172

Patel, V., Nolan, I. T., Morrison, S. D. and Fosnot, J. (2020) 'Visiting Subinternships in Wake of the COVID-19 Crisis: An Opportunity for Improvement', *Annals of Plastic Surgery,* 85(2S Suppl 2), pp. S153-S154. https://doi.org/10.1097/SAP.0000000000002444

## Appendices

None.

## Declarations

The author has declared that there are no conflicts of interest.

## Ethics Statement

Ethics Approval was not sought or required for this work as it is a "Personal view or opinion piece", not a research experiment.

## External Funding

This article has not had any External Funding

