# Supplementary material for: Recommendations for Medical Students Completing Virtual Rotations: Lessons Learned from Our Experience During the COVID-19 Pandemic
Source: MedEdPublish (2016). 2021 Feb 4;10:36. [Version 1] doi: 10.15694/mep.2021.000036.1 (PMC10939607; doi:10.15694/mep.2021.000036.1)
Supplement: Peer review history

# Reviewer report: Reviewer response for version 1

**DOI:** 10.21956/mep.18903.r27010

**Published:** 2021-07-09

**Authors:** John Sandars

**Affiliations:** 1 Edge Hill University

This review has been migrated. The reviewer awarded 4 stars out of 5 An essential read!This short opinion piece has several important messages that are highly relevant to a global readership in medical education.

Reviewer Expertise:

NA

No decision status is available

# Reviewer report: Reviewer response for version 1

**DOI:** 10.21956/mep.18903.r27009

**Published:** 2021-05-16

**Authors:** Madalena Folque Patricio

**Affiliations:** 1 School of Medicine, University of Lisbon

This review has been migrated. The reviewer awarded 5 stars out of 5 Congratulations on the paper. I was very pleased to see two students as authors.The topic is important to provide recommendations for online rotations implemented to address the limitations imposed by COVID-19. Let me start by highlighting some aspects of the list of recommendations: • The discussion about expectations, namely on what students can expect from the rotations as well as what is expected from them, so that they ‘can make the most’ of what is offered• The importance of having students actively involved in pre-reading and preparing for meaningful discussion during the sessions. This is extremely important not only to make the most of the specific sessions they will attend, but also to develop students’ responsibility for their own learning and the acquisition of lifelong learning skills. Teachers have a key role to play here in guiding students to the appropriate resources• The opportunities to have students in contact with research to increase their understanding of clinical medicine and facilitate critical thinking and critical appraisal • The acquisition/development of technical skills in a situation where face-to-face teaching is not allowed• The opportunity to develop students’ presentation skills a key communication competence as a student as a future Doctor.• The importance given to ‘Professionalism’, namely the importance given to the right attitude during online sessions. All these advices are important for those who want to implement this type of rotations. In terms of suggestions for improvement, I would have liked to have seen more information about the training of technical skills, because this is undoubtedly the most difficult aspect when offering similar rotations. In addition to the useful bibliography, the authors advised the installation of a second device focused on the technical skills station, to the device focused on the student, but some more guidance/details on how these virtual rotations were implemented, would be welcome.I would also like to have seen the results of the evaluation of these rotations because it would be important to get not only the seven recommendations but also the views from students and teachers, on the main strengths and difficulties felt. This would allow comparing student feedback with feedback from teachers, whose views on potentials and difficulties are expected to be more comprehensive. From the counterpoint to these two sources, other recommendations may emerge on how to improve future rotations. Finally, I would like to thank the authors for this useful paper on virtual rotations.

Reviewer Expertise:

NA

No decision status is available
